# Supplementary figures and images for: A Virus Hosted in Malaria-Infected Blood Protects against T Cell-Mediated Inflammatory Diseases by Impairing DC Function in a Type I IFN-Dependent Manner
Source: mBio. 2020 Apr 7;11(2):e03394-19. doi: 10.1128/mBio.03394-19 (PMC7157782; doi:10.1128/mBio.03394-19)

**A**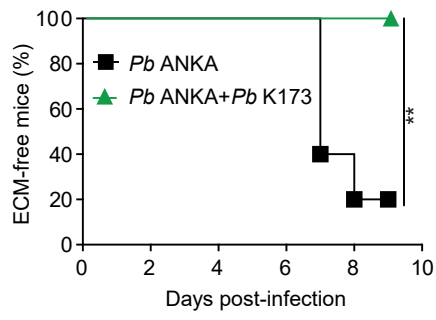**B**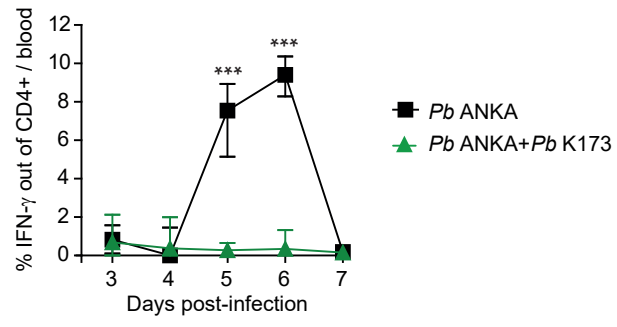

Supplement: FIG S1 [file mBio.03394-19-sf001.pdf]

Sup Figure 2

A

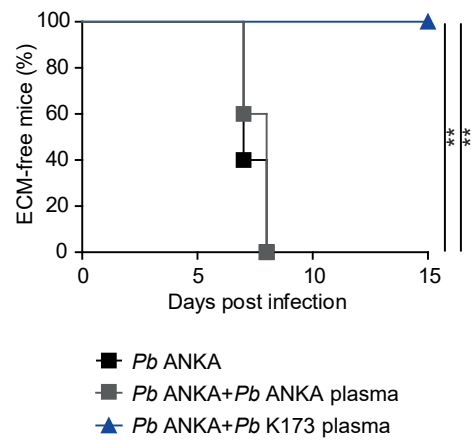

B

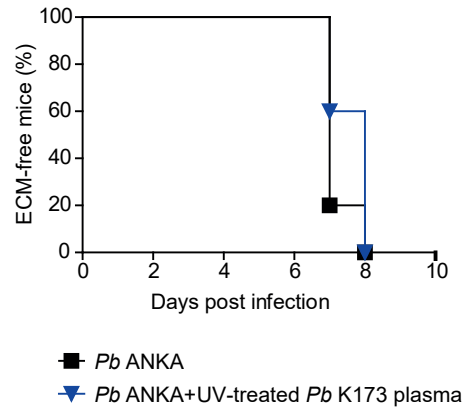

Supplement: FIG S2 [file mBio.03394-19-sf002.pdf]

Sup Figure 3

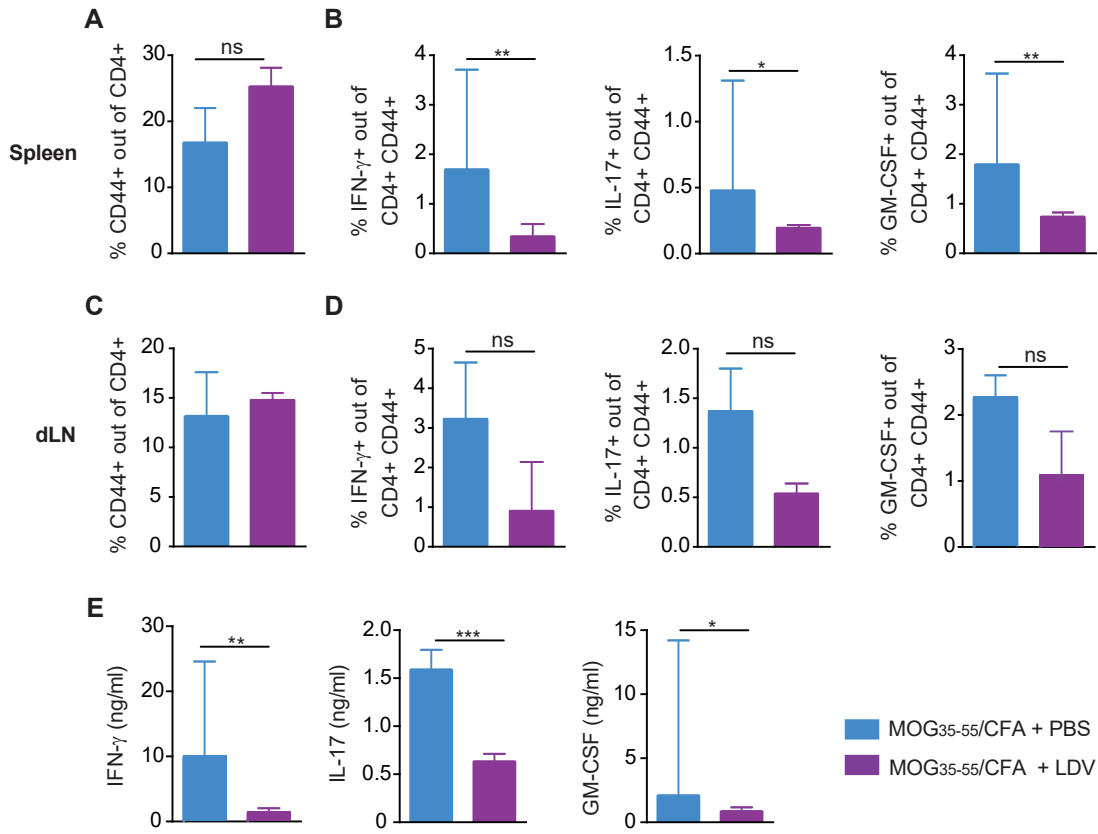

Supplement: FIG S3 [file mBio.03394-19-sf003.pdf]
